# Novel compound heterozygous MVK variants cause early-onset mevalonic aciduria in a Chinese infant

**DOI:** 10.3389/fped.2026.1887937

**Published:** 2026-07-15

**Authors:** Na Li, Weining Li, HongXia Zhang, Xingcui Wang

**Affiliations:** 1 Dermatology Department, Jinan Central Hospital, Jinan, Shandong, China; 2 Department of Dermatology, The Affiliated Hospital of Shandong University of Traditional Chinese Medicine, Jinan, Shandong, China; 3 Department of Rheumatology and Immunology, Children's Hospital Affiliated to Shandong University (Jinan Children's Hospital), Jinan, Shandong, China

**Keywords:** Chinese infant, mevalonate kinase deficiency, mevalonic aciduria, MVK, novel variants

## Abstract

Mevalonate kinase deficiency (MKD) is a rare autosomal recessive autoinflammatory disorder caused by mevalonate kinase (*MVK*) gene mutations, with phenotypes ranging from mild hyper-IgD syndrome (HIDS) to severe mevalonic aciduria (MA). Here, we report a 7-month-old Chinese male infant presenting with neonatal-onset MA. The proband presented with growth retardation, intractable diarrhea, recurrent fever, generalized rash, progressive hepatosplenomegaly, and persistent systemic inflammation. Laboratory tests revealed marked leukocytosis, elevated C-reactive protein, hyper-IgD and increased urinary mevalonic acid. Whole-exome sequencing identified two novel compound heterozygous *MVK* variants: c.64G > A (p.Val22Met) and c.1063G > C (p.Ala355Pro). Both variants were absent in global population databases. In silico analyses, including cross-species conservation, structural modeling, and pathogenicity prediction, confirmed significant structural perturbations. The patient achieved sustained remission with canakinumab. This is the first worldwide report of the *MVK* c.1063G > C variant causing severe MA, expanding the *MVK* mutational spectrum in Chinese populations.

## 1 Introduction

Mevalonate kinase deficiency (MKD, OMIM 260920/610377) is a rare monogenic autoinflammatory disease inherited in an autosomal recessive pattern, caused by loss-of-function mutations of *MVK* gene located on chromosome 12q24.11 (1). The encoded mevalonate kinase (MK) is a rate-limiting enzyme in the mevalonate pathway, catalyzing the phosphorylation of mevalonic acid to 5-phosphomevalonate, a key step in cholesterol and isoprenoid biosynthesis (2). Loss-of-function mutations impair MK enzymatic activity, resulting in massive accumulation of mevalonic acid and its metabolites, which triggers over-activation of the NLR family pyrin domain containing 3 (NLRP3) inflammasome, excessive secretion of pro-inflammatory cytokines, and systemic autoinflammatory responses (3).

Clinically, MKD is classified into two main phenotypes based on residual MK activity: mild hyper-IgD periodic fever syndrome (HIDS) and severe mevalonic aciduria (MA) (4). HIDS is characterized by recurrent febrile episodes, cutaneous rash, lymphadenopathy, abdominal pain, and arthralgia, with residual MK activity ranging from 0.5% to 20%, usually presenting in late infancy or early childhood (5, 6). In contrast, MA represents the severe end of the clinical spectrum, with residual MK activity less than 0.5%, manifesting with neonatal or early-infantile onset, severe growth retardation, intractable diarrhea, progressive hepatosplenomegaly, persistent systemic inflammation, neurological impairment, and high early mortality (7). Previous cohort studies have indicated that more than 90% of *MVK* pathogenic variants are private family-specific mutations, with few recurrent founder variants globally (8, 9).

To date, more than 320 *MVK* pathogenic variants have been documented worldwide, predominantly missense mutations, followed by splicing variants, nonsense mutations, and small indels (10, 11). The p.V377I (c.1129G > A) is the most common worldwide founder variant, especially in Northern Europeans (12). p.I268T (c.803T > C) is the second hotspot associated with intermediate-severe disease (13, 14). However, early-onset MA has been reported less frequently in Chinese populations, frequently misdiagnosed as sepsis.

Here, we report a 7-month-old Chinese infant with severe neonatal-onset MA, carrying compound heterozygous *MVK* variants: c.64G > A (p.Val22Met) and c.1063G > C (p.Ala355Pro). Both variants were novel in MA patients. Comprehensive bioinformatics and structural analyses confirmed severe functional impairment. The patient responded dramatically to canakinumab. This study expands the mutational spectrum of MKD in East Asian populations, provides evidence for early differential diagnosis of neonatal autoinflammatory disorders, and deepens our understanding of genotype-phenotype correlations in severe MA.

## 2 Materials and methods

### 2.1 Ethical approval and patient information

This study was approved by the Ethics Committee of the Affiliated Children's Hospital of Shandong University (No.SDFE-IRB/P2025012), and conducted according to the Declaration of Helsinki Principles. Informed consent from the parents of the child was obtained before clinical and laboratory examinations. Peripheral blood samples were collected from the patient and his healthy parents. The index patient was a 7-month-old Chinese male infant, the second child of non-consanguineous healthy parents from Shandong Province, Eastern China. The family history indicated that the proband's elder brother had recurrent fever, poor growth, and died at 3 months of age.

### 2.2 Clinical data collection

Longitudinal clinical data were collected from neonatal admission to follow-up. Laboratory tests included complete blood count, inflammatory markers [C-reactive protein (CRP), serum amyloid A (SAA), erythrocyte sedimentation rate (ESR), ferritin], serum immunoglobulin profiles (IgA, IgM, IgD), liver and renal function, urinary organic acid analysis via gas chromatography-mass spectrometry (GC-MS) for mevalonic acid quantification. Imaging examinations included chest computed tomography (CT), abdominal ultrasound, cranial magnetic resonance imaging (MRI), and cardiac ultrasound. Infectious screening tests, including pathogen PCR, serological assays, and autoimmune antibody panels, were performed to exclude infectious diseases and other autoimmune disorders.

### 2.3 Whole-exome sequencing and variant analysis

Peripheral blood samples were collected from the proband and his parents. Genomic DNA was isolated from leukocytes using a commercial DNA extraction kit (Tiangen, Beijing, China). Whole-exome capture and sequencing were performed on the NovaSeq 5,000 platform (Illumina, USA) using a human exome probe set (MyGenostics, Beijing, China). The average sequencing depth reached 100×, with over 95% of target regions covered at ≥10×. Raw reads were aligned to the human reference genome (GRCh37/hg19) using the Burrows-Wheeler Aligner (BWA). Variants were filtered against population databases including the 1,000 Genomes Project, Exome Aggregation Consortium (ExAC), and Exome Variant Server (EVS), to exclude common polymorphisms with allele frequencies >5%. Functional effects were predicted using PolyPhen-2, SIFT, and MutationTaster. Splicing-related variants were evaluated with Human Splicing Finder (HSF). Cross-species conservation was assessed via multiple sequence alignment using ClustalX. Three-dimensional protein structural modeling of wild-type and mutant proteins was generated with the SWISS-MODEL server. Variant interpretation followed the 2015 ACMG guidelines, with variants categorized as pathogenic, likely pathogenic, variant of uncertain significance (VUS), likely benign, or benign. Sanger sequencing was performed to confirm all candidate variants and verify parental segregation.

## 3 Results

### 3.1 Case description in our proband

**Figure 1:**
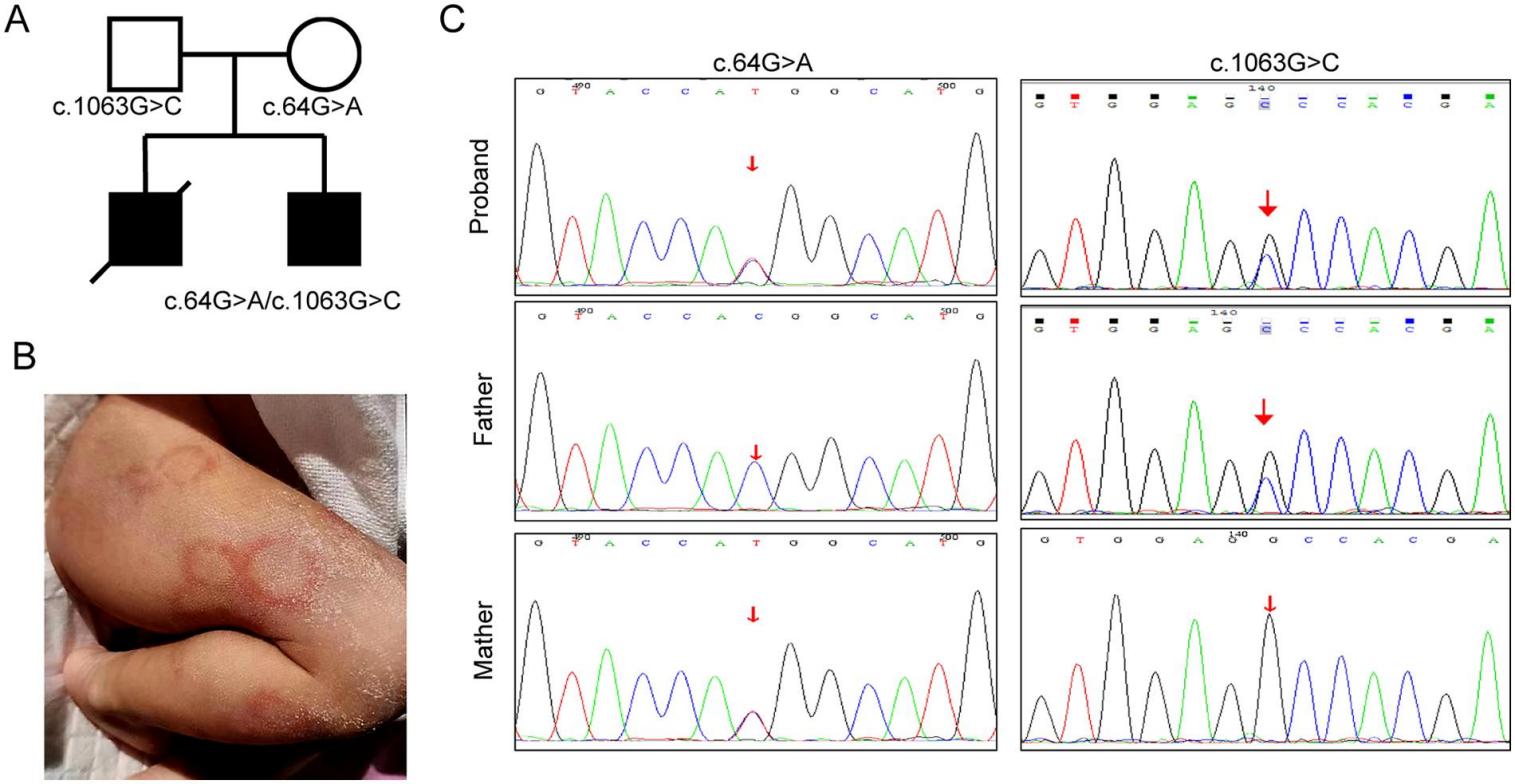
Early onset case of MKD harbouring novel *MVK* genotype. **(A)** Pedigree and familial segregation of the *MVK* alleles with the affected individual marked in black and unaffected in white. **(B)** Widespread erythematous, non-blanching rash at 6 months of age. **(C)** Sanger sequencing traces showing c.64G > A and c.1063G > C variants in the patient and parents.

The male proband was delivered via cesarean section at 38 weeks gestation with a birth weight of 2.15 kg. The parents were healthy and non-consanguineous, with a family history: the elder brother died at 3 months of age with recurrent fever and failure to thrive (Figure 1A). At one month of age, he presented with growth retardation and intractable chronic diarrhea. Physical examination revealed poor nutritional status, generalized pallor, scattered erythematous maculopapular rashes on the extremities, and hepatosplenomegaly (liver 3.0 cm, spleen 1.5 cm below the costal margins) with abdominal venous distension. Initial laboratory tests showed mild anemia and elevated inflammatory markers, accompanied by abnormal autoimmune profiles. Chest CT demonstrated bilateral lower lobe inflammatory infiltrates. Early differential diagnoses included malnutrition, enteritis, primary immunodeficiency, and lysosomal storage disorders.

Between 3 and 5 months of age, the patient developed recurrent periodic fever (peak temperature up to 39.0 °C, every 4–6 weeks), generalized maculopapular rash (Figure 1B), persistent diarrhea. Abdominal ultrasound at 5 months of age confirmed progressive pathological hepatosplenomegaly: the liver edge extended 4.2 cm below the right midclavicular costal margin, the spleen thickness reached 2.2 cm, and the splenic tip descended 3.6 cm beneath the left costal margin. Lymphadenopathy was also detected. Episodes were unresponsive to antibiotics and corticosteroids, relapsing rapidly after treatment cessation. Respiratory symptoms included recurrent cough and mixed infection with *Mycoplasma pneumoniae* and human metapneumovirus. Persistent leukocytosis, neutrophilia, thrombocytosis, and elevated inflammatory markers were noted throughout this period (Table 1). Laboratory tests revealed persistent mild anemia, thrombocytosis, markedly elevated IgD (197 mg/L, normal range: 0–100 mg/L), GC-MS confirmed significantly increased urinary mevalonic acid (17.6 μg/mg creatinine), consistent with mevalonate kinase pathway dysfunction. Subclinical central nervous system involvement was detected on cranial MRI, showing mild widening of left frontal extra-axial spaces and delayed myelination without gross structural malformations, consistent with mild neurodevelopmental sequelae specific to early-onset MA. A small patent foramen ovale was incidentally identified as an isolated congenital cardiac developmental variant.

**Table 1:** Clinical and laboratory parameters of the patient.

Parameter	Value	Normal range	Interpretation
White blood cell count, cells/L	24.8×109	5.0–14.0 × 109	↑
Neutrophil count, cells/L	11.77 × 109	0.8–6.5 × 109	↑
Neutrophil, %	53.1	15.0–45.0	↑
Lymphocytet, %	37.8	20.0–40.0	N
Hemoglobin, g/L	95	110–140	↓
Platelets (×10⁹/L)	620	150–450	↑
Erythrocyte sedimentation rate, mm/h	46	0–23	↑
C-reactive protein, mg/dL	81.50	0–10	↑
Serum amyloid A, mg/L	>320.0	0–10	↑
IgA, g/L	0.353	0.15–1.09	N
IgD, mg/L	197	0–100	↑
IgM, g/L	3.390	0.65–2.01	↑
ALT, U/L	78	5–60	↑
AST, U/L	89	15–60	↑
Urine mevalonic acid, ug/mg (Intermittent period)	17.6	0–5	↑

N, normal; ↑, elevated; ↓, decreased.

Following genetic confirmation of compound heterozygous MVK variants at 6 months of age, the patient received anti-IL-1β targeted therapy with canakinumab (37.5 mg, subcutaneous every 4 weeks). After the first administration, fever, rash, and diarrhea resolved within days. Inflammatory markers normalized within two weeks, hepatosplenomegaly gradually regressed. No adverse events were observed during treatment.

### 3.2 Genetic findings

Whole-exome sequencing identified two compound heterozygous missense variants of the MVK gene in the proband, including c.64G > A (p.Val22Met) and c.1063G > C (p.Ala355Pro) (Figure 1C). Both variants were novel and unreported in peer-reviewed publications and public databases including OMIM, UCSC Genome Browser, HGMD, dbSNP, 1,000 Genomes, ExAC, and gnomAD. Population frequency analysis revealed extremely low allelic frequencies of 0.0000041 for c.64G > A and 0.000641 for c.1063G > C in the gnomAD database, with both variants absent from the 1,000 Genomes and ExAC cohorts. The missense variant c.64G > A (p.Val22Met) caused an amino-acid substitution from hydrophobic valine (Val) to polar methionine (Met) at residue 22, which is located within the well-recognized mutational hotspot region of the N-terminal hydrophobic core domain of mevalonate kinase. The other missense variant c.1063G > C (p.Ala355Pro) led to a substitution of non-polar alanine (Ala) with rigid proline (Pro) at residue 355, a critical site within the ATP-binding catalytic pocket. Sanger sequencing validated both variants in the proband and confirmed parental segregation: the mother was heterozygous for c.64G > A, while the father carried the heterozygous c.1063G > C variant, consistent with an autosomal recessive inheritance pattern (Figure 1C). According to the 2015 American College of Medical Genetics and Genomics (ACMG) variant interpretation guidelines, c.64G > A and c.1063G > C were classified as variants of uncertain significance.

### 3.3 Pathogenicity validation

**Figure 2:**
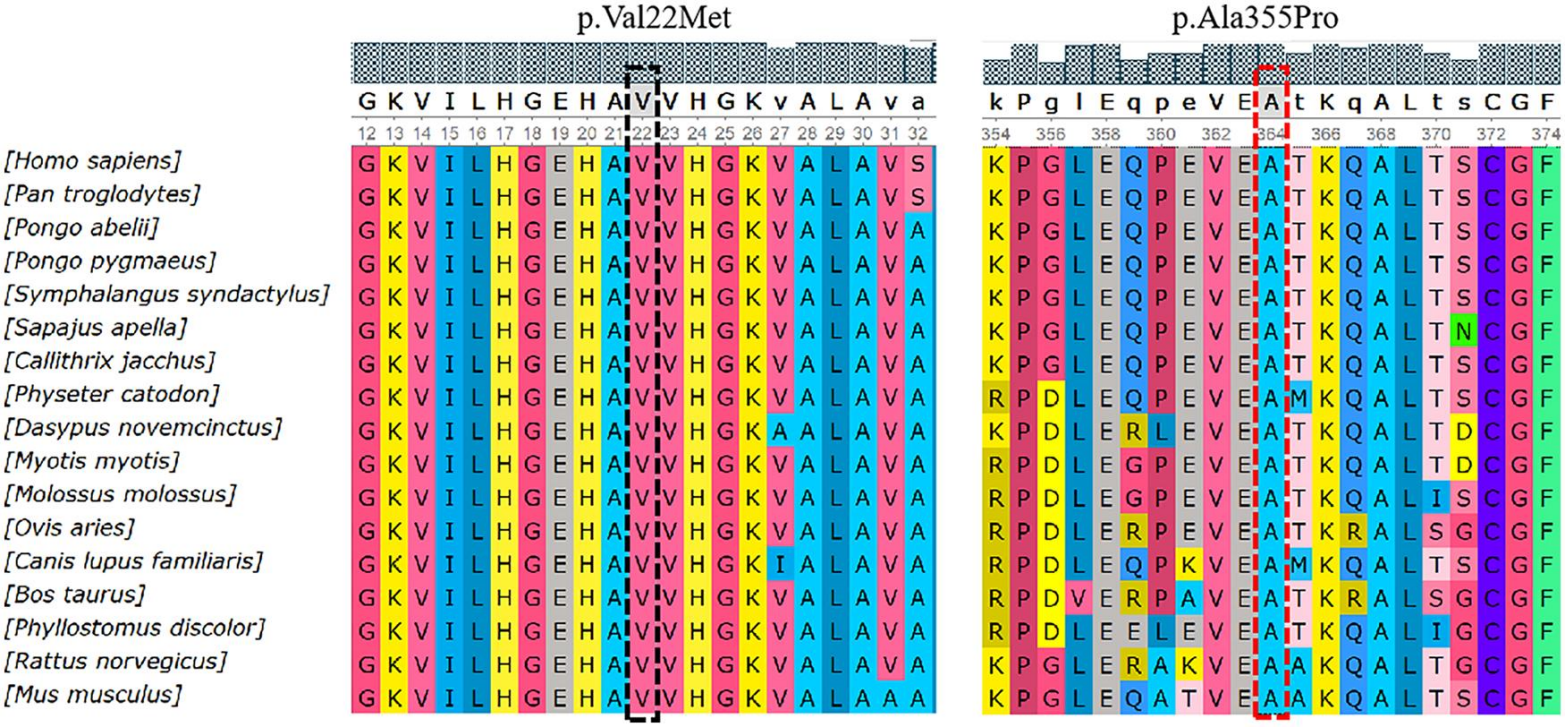
Cross-species multiple-sequence alignment of MVK protein residues Val22 and Ala355. Multiple-sequence alignment across mammalian species demonstrates strict evolutionary conservation of residue Val22 (left panel, dashed black box) and Ala355 (right panel, dashed red box).

Cross-species conservation analysis, multiple bioinformatic functional predictions, and three-dimensional protein structural modeling were performed sequentially to evaluate the pathogenic potential of the two identified MVK variants. Cross-species multiple-sequence alignment across 17 mammalian species revealed that both residues Val22 and Ala355 were strictly evolutionarily invariant across all examined taxa (Figure 2), indicating their indispensable roles in maintaining the structural integrity and catalytic function of mevalonate kinase.

Comprehensive in silico pathogenicity prediction using four independent algorithms demonstrated distinct functional impacts of the two variants (Table 2). For p.Val22Met, all tools uniformly predicted deleterious effects, including SIFT (damaging), PolyPhen-2 (probably damaging), MutationTaster (disease-causing), with a high REVEL score of 0.92 exceeding the pathogenic threshold of 0.5. For p.Ala355Pro, PolyPhen-2 (probably damaging), MutationTaster (disease-causing), and a high REVEL score of 0.96 strongly supported its pathogenic nature.

**Table 2:** Effect on protein function of the novel MVK variations predicted by SIFT, polyPhen-2, mutationTaster and REVEL.

Gene	Variant	Variant type	GnomAD	SIFT		PolyPhen-2		Mutation Taster		REVEL	
				Score	P	Score	P	Score	P	Score	P
MVK	c.64G>A	Missense	0.0000041	0	D	1	PD	1	DC	0.92	D
MVK	c.1063G>C	Missense	0.000641	0.101	T	0.987	PD	1	DC	0.96	D

P, Prediction; D, Damaging; PD, Probably_damaging; DC, Disease_causing; T, Tolerated.

**Figure 3:**
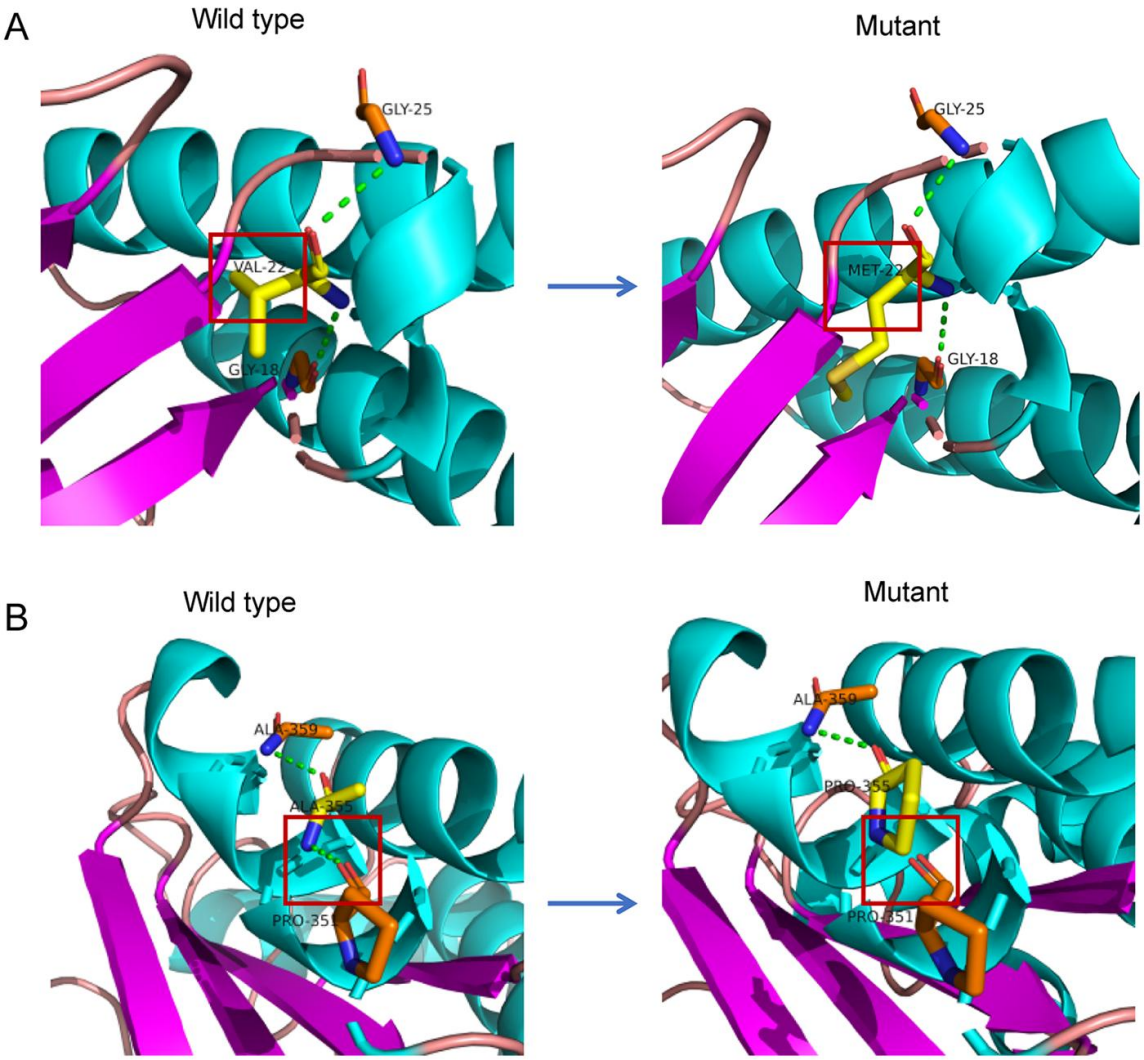
Three-dimensional structural modeling of wild-type (WT) and mutant MK proteins based on template PDB: 2r3v.1.A, illustrating variant-induced structural alterations. **(A)** Local structural view of the N-terminal hydrophobic core in WT and p.Val22Met mutant MK. **(B)** Local structural view of the ATP-binding pocket in WT and p.Ala355Pro mutant MK. The c.1063G > C (p.Ala355Pro) substitution at residue 355 reduces the number of local hydrogen bonds from two to one.

Three-dimensional structural modeling of human MK was performed using the SWISS-MODEL server, with the wild-type (WT) template PDB: 2r3v.1.A. The MK protein consists of an N-terminal hydrophobic domain, a mevalonate-binding pocket, and an ATP-binding catalytic core. For the c.64G > A (p.Val22Met) variant, structural comparison showed that substitution of valine to methionine at residue 22 did not alter the local hydrogen-bond network but disrupted intramolecular hydrophobic packing within the N-terminal hydrophobic core, leading to destabilization of local protein folding (Figure 3A). Abnormal folding may destabilizes protein spatial conformation and reduces steady-state content of functional MK enzyme. For the c.1063G > C (p.Ala355Pro) variant, replacement of alanine with proline at residue 355 reduced the number of local hydrogen bonds from two to one, induced a helix kink in the α-helix of the ATP-binding pocket, and impaired substrate binding (Figure 3B). Helix kink plus reduced local hydrogen bonds may disrupt ATP-substrate binding interface, drastically lowering catalytic efficiency of MK. Collectively, these structural disruptions are predicted to affect MK enzymatic function.

## 4 Discussion

In this study, we systematically characterized a Chinese infant diagnosed with severe mevalonic aciduria, identified two novel compound heterozygous *MVK* pathogenic variants, validated their pathogenicity via comprehensive in silico analyses, and analyzed longitudinal clinical manifestations and therapeutic responses. This work expands the mutational spectrum of MKD in Chinese populations and provides critical insights into early diagnosis and targeted management of neonatal-onset MA.

We report two novel compound heterozygous *MVK* variants (c.64G > A/ c.1063G > C) in a Chinese infant. The variant c.64G > A (p.Val22Met) is located in the N-terminal hydrophobic core domain of MVK, a well-recognized mutational hotspot for severe MKD. This variant has been previously reported in patients with disseminated superficial porokeratosis (DSP), but it has never been documented in classic mevalonic aciduria (MA) or severe systemic MKD (15, 16). Porokeratosis (PK) is a rare autosomal dominant keratinization disorder caused by dominant germline or somatic mutations in the mevalonate pathway genes, including MVK (17). The N-terminal hydrophobic core (residues 1–50) is essential for maintaining MK protein stability, dimerization, and membrane association. The substitution p.Val22Met disrupts hydrophobic packing, destabilizes local folding, and impairs enzyme solubility. In porokeratosis, this variant causes partial enzyme dysfunction, predominantly affecting keratinocyte metabolism. Notably, our patient exhibited generalized maculopapular rash, a cutaneous phenotype overlapping with porokeratosis. This suggests that p.Val22Met may predispose to cutaneous inflammation, suggesting a potential novel genotype-cutaneous phenotype correlation in MKD, which requires further validation in larger cohorts.

The variant c.1063G > C (p.Ala355Pro) is globally novel and absent from all published MKD cohorts and public databases. It maps to the highly conserved ATP-binding catalytic pocket of MVK, a domain essential for substrate binding and enzymatic catalysis. Residue Ala355 is strictly conserved across 15 mammalian species, confirming its functional indispensability. The Ala355Pro substitution introduces a rigid proline, which induces a helix kink and reduces local hydrogen bonds from two to one, weakening interactions within the catalytic pocket, predicting significant impairment of MK activity. Globally, ATP-binding pocket mutations (e.g., p.V310M, p.A334T) are strongly associated with severe MA, neurological impairment, and ophthalmic involvement (1, 18, 19). Our novel variant extends this hotspot, confirming that catalytic-domain mutations confer the most severe MKD phenotypes. The proband's compound heterozygous genotype (p.Val22Met/p.Ala355Pro) represents a novel severe combination, this dual-domain disruption results in synergistic functional impairment, explaining the neonatal onset, persistent inflammation, and multi-organ involvement.

Our patient exhibited a full longitudinal clinical spectrum of MA, starting with neonatal-onset growth retardation and chronic diarrhea, progressing to recurrent fever, rash, hepatosplenomegaly, and persistent inflammation, mimicking bacterial sepsis. Neonatal-onset MA is extremely rare, with most global cases reported in European populations (11, 20, 21). In Chinese clinical practice, neonatal mevalonic aciduria (MA) is frequently misdiagnosed as neonatal sepsis, congenital infections, or primary immunodeficiency, leading to delayed diagnosis and poor prognosis. Key clinical features include persistent recurrent fever that is unresponsive to routine antibacterial therapy, unexplained persistent thrombocytosis, markedly elevated acute-phase inflammatory markers, elevated serum IgD levels, progressive hepatosplenomegaly, intractable chronic diarrhea, and failure to thrive. Definitive diagnosis ultimately relies on elevated urinary mevalonic acid and the identification of pathogenic biallelic MVK variants through genetic testing. These diagnostic clues can assist clinicians in distinguishing MA from neonatal sepsis, congenital infectious diseases, and primary immunodeficiency during early clinical screening. Consistent with previous cohort studies of MA, our patient presented with thrombocytosis, markedly elevated IgD, and anemia, which are well-recognized laboratory hallmarks of MKD and reflect chronic IL-1-mediated autoinflammation (19). Anti-IL-1β targeted therapy with canakinumab achieved clinical remission in our patient, confirming that IL-1β over-activation is the core pathogenic mechanism of MA, and validating anti-IL-1 agents as first-line therapy for severe MA.

The proband originated from a family with a previously deceased elder sibling, who presented with recurrent fever, progressive hepatosplenomegaly, and failure to thrive,clinical features highly suggestive of severe neonatal-onset MA, without confirmatory genetic testing on the deceased child. This family history underscores the critical importance of recognizing familial recurrence risk in infants with unexplained early-onset autoinflammation or infantile death of unknown cause. For families with a history of suspected or confirmed MKD, systematic genetic counseling and carrier screening are essential to guide reproductive decisions and prevent recurrent severe disease. Parental carrier testing is strongly recommended, as heterozygous parents of affected children are at a 25% risk of having another affected child in subsequent pregnancies. Prenatal diagnosis via chorionic villus sampling or amniocentesis enables early identification of affected fetuses and supports informed pregnancy management. This case highlights that unexplained infant death in high-risk families should prompt consideration of monogenic autoinflammatory disorders, including MKD, and initiate timely genetic evaluation and counseling to reduce recurrence risk.

This study has several limitations: functional validation of MK enzymatic activity via *in vitro* expression assays was not performed due to technical constraints, and long-term follow-up data for neurological development are still ongoing. Future studies will include *in vitro* enzymatic activity testing of mutant MK proteins to further validate pathogenicity, and long-term clinical follow-up to assess neurological and developmental outcomes of the patient under targeted therapy. Furthermore, large-scale cohort studies of Chinese MKD patients are required to further define the mutational spectrum and genotype-phenotype correlations in East Asian populations.

## 5 Conclusion

We report two novel compound heterozygous missense variants [c.64G > A [p.Val22Met] and c.1063G > C [p.Ala355Pro]] in the *MVK* gene in a Chinese infant with early-onset mevalonic aciduria. Comprehensive in silico analyses validated that both variants disrupt critical functional domains of mevalonate kinase. The patient exhibited typical neonatal-onset MA manifestations and achieved sustained remission with anti-IL-1β targeted therapy. This study expands the mutational spectrum of MKD in Chinese populations, highlights the clinical value of early genetic screening for infants with unexplained autoinflammation, and provides evidence for genetic counseling of high-risk MKD families.

## Data Availability

The original contributions presented in the study are included in the article/Supplementary Material, further inquiries can be directed to the corresponding author.
